# Diagnostic Values of Serum Inflammatory Biomarkers after Hip and Knee Arthroplasty in Patients with Periprosthetic Joint Infection

**DOI:** 10.3390/healthcare12151511

**Published:** 2024-07-30

**Authors:** Bogdan-Axente Bocea, Mihai-Dan Roman, Nicolas Catalin Ionut Ion, Sorin Radu Fleaca, Cosmin-Ioan Mohor, Darius Alexandru Popa, Romeo-Gabriel Mihaila

**Affiliations:** 1 Faculty of Medicine, Lucian Blaga University of Sibiu, Str. Lucian Blaga, Nr. 2A, 550169 Sibiu, Romania; bogdanaxente.bocea@ulbsibiu.ro (B.-A.B.); nicolascatalinionut.ion@ulbsibiu.ro (N.C.I.I.); radu.fleaca@ulbsibiu.ro (S.R.F.); cosmin.mohor@ulbsibiu.ro (C.-I.M.); romea.mihaila@ulbsibiu.ro (R.-G.M.); 2 County Clinical Emergency Hospital, 550245 Sibiu, Romania; dariusapopa@yahoo.com

**Keywords:** THA, TKA, ESR, fibrinogen, CRP, PJI

## Abstract

One of the complications after total hip arthroplasty (THA) or total knee arthroplasty (TKA) is periprosthetic joint infection (PJI). Numerous studies have been performed to explore the value of biological parameters in the early identification of infection rates after THA and TKA. This study investigates alterations in inflammatory markers associated with PJI. This retrospective study focused on a cohort of patients with hip and knee arthroplasty treated between 2016 and 2022. CRP, ESR, and fibrinogen were observed preoperatively, on days one, three, six, and twenty-one postoperatively. From a total of 4076 THA and TKA performed during this period, 62 patients were identified with periprosthetic infections. We also identified the pathogens responsible for infections in order to assess if asymptomatic preoperative infections were involved in PJI. In patients with acute infections following TKA, days one and three postoperative recorded a CRP value below the expected range. The value of CRP in patients with early infection after THA was significantly increased on day six postoperative. ESR and fibrinogen values were not statistically significantly correlated with early PJI. The CRP level in acute PJI shows different patterns than those shown in the literature.

## 1. Introduction

Total hip replacement and total knee replacement have been routinely conducted since the 1970s, with over one million procedures performed in the United States in 2009 [1]. Hip arthroplasty, while common, is linked to a mortality rate ranging from 1% to 2% and a notable incidence of complications, periprosthetic infections being the most challenging ones [2]. Consequently, the absolute contraindication for this procedure is the presence of chronic or active infections in the body or joint, while relative contraindications encompass rapidly progressing neurological diseases or pathologies leading to bone destruction or functional failure of the abductor muscles.

One significant complication after surgery is infection, which can be either superficial or deep, with the latter being more severe. The incidence of deep infections varies between 1% and 10%, a range influenced by preventive measures such as preoperative antibiotic administration.

The rising frequency of these surgeries has contributed to a heightened incidence of infections. Concurrently, the escalating prevalence of antibiotic resistance emphasizes the critical role of early intervention in infection prevention and treatment. Timely diagnosis within the initial 3–4 weeks frequently allows for a conservative approach; however, delayed identification may necessitate prosthesis removal because of the formation of antibiotic-resistant biofilms by the microorganisms involved [3].

Periprosthetic infections, albeit infrequent with an incidence of 1–2%, pose a life-threatening challenge and contribute to 15% of hip replacement failures [4]. Treatment modalities are contingent on the extent of tissue damage and the patient’s overall condition. Antibiotic therapy, irrigation, and tissue debridement may be sufficient for minor infections. However, more severe cases may necessitate one-stage or two-stage revisions involving implant removal, spacer implantation, antibiotic cement, or, in extreme scenarios, the Girdlestone procedure, with a high impact on patient well-being and limb function [5,6].

Research has been directed towards the utilization of inflammatory biomarkers for early detection of complications in hip and knee replacement surgeries [7].

Recent investigations have examined alterations in erythrocyte sedimentation rate (ESR) and C-reactive protein (CRP) levels as potential early detection markers following surgery. The duration of elevated CRP levels post-surgery exhibits variability dependent on the type of surgical procedure, with values typically returning to baseline within three weeks after hip surgery [8]. In contrast, the ESR peaks on the fifth day post-surgery, normalizing after approximately ninety days. Notably, a comprehensive study encompassing both total hip arthroplasty (THA) and total knee arthroplasty (TKA) surgeries revealed no significant disparities in the postoperative changes observed in these parameters [9].

Based on the literature, there is an immediate postoperative increase in CRP levels, with the maximum value typically recorded on the third day. Some studies report this peak within the first forty-eight hours, with complete normalization observed between fourteen and forty-two days [10,11]. The literature establishes that the peak value of the ESR following orthopedic surgery typically occurs on the fifth day. The gradual normalization trend in this parameter allows for a return to normal limits within ninety days post-surgery [12]. The analysis of fibrinogen variation reveals that this trauma marker exhibits an initial increase in the first hours after injury, attaining its peak value on the third and fourth day, respectively. It demonstrates a tendency to revert to its baseline within the first week post-intervention [13].

This study aims to investigate the potential utility of monitoring acute-phase proteins post-surgery for early detection of periprosthetic infections, an inquiry that has garnered increasing attention within the medical literature. The objective is to enhance the early diagnosis of septic complications, improving treatment outcomes and potentially reducing hospitalization costs. Simultaneously, this study explores the microbial pathogens associated with these infections.

## 2. Materials and Methods

This single-center, retrospective, observational study was conducted at the Sibiu Emergency Hospital in Romania. Before the inclusion of patients in this study, the research protocol underwent a thorough review and received approval from the institutional review board. A standardized system was uniformly applied to all study participants, specifically targeting patients who developed infections following total or partial hip arthroplasty and total knee arthroplasty. The determination of infection presence was established through preoperative, intraoperative, and postoperative wound sampling.

The retrospective study cohort comprised patients who underwent either partial hip replacement, THA, or TKA within the Orthopedics and Traumatology Department of the Sibiu Emergency County Clinical Hospital from 2016 to 2022. This group specifically included patients in whom a postoperative superficial or deep infection was identified. Infections were identified in 62 patients out of a total of 4076 hip and knee arthroplasties.

This study analyzed the fluctuations in CRP, fibrinogen, and the ESR among patients with periprosthetic infections, aiming to provide supplementary insights.

Inflammatory samples (blood samples to determine the CRP, fibrinogen, ESR), urine culture [14], and pharyngeal exudate [15] were collected from all patients on the day before the surgery, with additional samples from patients with a femoral neck fracture.

Inflammatory samples were collected on the first day after surgery, the third day after surgery, and the sixth day after surgery from all patients. On day six, the patients were discharged according to the ward protocol if no other complications occurred during the postoperative period. During TKA surgery, a tourniquet was used, and no urinary catheter was used during surgery; after surgery, no drainage was used, and the patients were mobilized immediately postoperative, with wound dressing days 1, 3, and 6 after surgery. For patients who underwent hip replacement following femoral neck fractures, urine culture and pharyngeal exudate samples were collected to assess asymptomatic infections at these sites.

After patient discharge, 21 days postoperatively, the patients were called in for suture removal, and additional inflammatory samples were obtained.

The exclusion criteria were patients with a history of pathologies that influenced inflammatory samples and for whom the reference level no longer corresponded. The patients who failed to show up for their day 21 check-up were also excluded.

The patients were categorized based on the type of periprosthetic infection, i.e., acute or chronic, following the criteria outlined in the “Pocket Guide to Diagnosis & Treatment of Periprosthetic Joint Infection of the PRO-IMPLANT Foundation, Berlin, Germany (coordinated by N. Renz and A. Trampuz)” [16]. Acute infections were defined as those occurring within the first four weeks after surgery, while chronic infections manifested more than four weeks post-surgery. Among the sixty-two patients, thirteen were identified with acute infections, and thirty-two exhibited chronic infections.

For the statistical analysis of the obtained results, we used SPSS Statistic 28.0 Software. The quantitative parameters were described as means ± standard deviations, and the qualitative parameters were expressed as frequencies or percentages. All tests were two-sided, and *p* values less than 0.05 were considered statistically significant [17].

## 3. Results

To observe the variation in inflammatory biomarkers in patients without an infection, we took a group of one hundred and fifty patients who underwent either cemented or uncemented total hip endoprostheses, along with total knee endoprostheses, during the period from 1 May 2018 to 31 August 2018, at the Orthopedics and Traumatology Department of the Sibiu Emergency County Clinical Hospital. The alterations in inflammatory biomarker levels are detailed in Table 1.

In our cohort of sixty-two patients, a comprehensive analysis of these parameters was conducted [17]. These patients underwent the following procedures: 20 patients underwent TKA, 28 patients underwent THR after osteoarthritis, and of the 14 patients with a femoral neck fracture, 10 of them underwent THA and 4 of them underwent partial hip replacement.

Regarding the ESR and fibrinogen, no significant changes were identified that could signal a potential infection. Variations in CRP levels displayed deviations from the expected norm in this analysis. So, we decided to focus only on the variations in postoperative CRP values.

Compared with these mean values obtained in this standard group, in our group of patients with postoperative infections, we obtained the following values (Table 2).

The observed changes exhibited distinct patterns based on the type of prosthesis implanted. Consequently, we stratified the patients into two groups as follows: those who underwent hip arthroplasty and those who underwent total knee arthroplasty.

In the context of hip arthroplasty patients, the observed changes were not readily apparent. On the first postoperative day, alterations were within the normal range, with the maximum value also attained on day three. However, in both chronic and acute infection cases, the values on day six did not exhibit the anticipated decrease. The baseline value from our previous dataset and the literature suggests an expected range of around 20–30 mg/L, with an average of 26.35 mg/L at six days in our prior data. In the group with infections, values around 40–50 mg/L were noted, and in some isolated instances of acute infection, the values even surpassed 100 mg/L.

Persistently elevated CRP values beyond the reference range were evident in all patients with acute infections after total hip arthroplasty, even twenty-one days post-surgery. The majority exhibited CRP values higher than the reference level on day six, with instances reaching 30.99 mg/L, 82.79 mg/L, and, in extreme cases, values of 364.85 mg/L or 167.70 mg/L. A subset of five patients exhibited slightly lower values, hovering around 15 mg/L, though still elevated compared with the normal range in this postoperative interval. Conversely, in patients with chronic infections after total hip arthroplasty, no substantial changes were observed 21 days post-surgery, with all maintaining CRP values akin to the preoperative levels (Table 3).

The differences in CRP values in acute and chronic infections were approximately the same between acute and chronic infections on days 1 and 3, which means we had approximately the same increase in both groups. The difference between batches on day 6 was much larger than before, showing a slower decrease in CRP in acute infection.

Following acute infection after TKA, discernible changes manifested from the first postoperative day. In patients with acute infection, the value on the initial postoperative day was notably lower than the standard observed in both our reference group and the literature. The average value on the first day post-arthroplasty, typically around 80 mg/L, contrasted with our observed average of approximately 35 mg/L, with all patients falling within the 30–40 mg/L range.

For patients with chronic infections, substantial changes were less apparent. Only one case presented a value of 141.72 mg/L on the first postoperative day, markedly higher than the normal range, and another case recorded a value of 24.12 mg/L, considerably lower than the standard. Subsequently, an increase peaked on days two to three, although these values were lower in patients with acute infections compared with the typical levels on these days. Our reference value from previous data was approximately 108 mg/L, consistent with values found in the literature. In patients with ongoing infections, the average CRP value ranged from 60–75 mg/L.

On day three postoperative, patients with chronic infections exhibited minimal changes. In the case with a value of 141.72 mg/L, it only increased marginally to 155.56 mg/L. On day six, both acute and chronic infection groups approached the normal reference level, which in our cohort was 65 mg/L. An important deviation from normal occurred in the case of chronic infection with a postoperative CRP value of 24.12 mg/L, showing only 9.48 mg/L at this juncture. On day twenty-one, minimal changes were observed, with values approximating the normal range (Table 4).

The difference in CRP values between patients with chronic and acute infection was quite large from day 1. On days 3 and 6, they were higher, but they were higher for chronic infections. Only towards day 21, the difference started to be smaller but still higher for chronic infections.

Among the fourteen patients subjected to emergency intervention following a femoral neck fracture, both urine cultures and pharyngeal exudates were systematically collected. Notably, twelve of these cases revealed the identification of microbes in either the urine culture or pharyngeal exudate, indicating an 85% prevalence of asymptomatic infection within this subgroup. The identified pathogens in urine culture included *Pseudomonas aeruginosa* (two cases), *Klebsiella pneumoniae* (five cases), *Proteus* (two cases), and *Enterobacter cloacae* (one case). In the pharyngeal exudate, fungi were most frequently encountered (seven cases), along with *Staphylococcus aureus* (two cases) and *Enterobacter cloacae* (one case, the same patient who also exhibited *Enterobacter cloacae* in the urine culture).

Of these 12 patients, 6 subsequently developed infections with the same germ that was identified in urine cultures or pharyngeal exudates.

Of particular note, one patient with a *Klebsiella pneumoniae* infection identified in the urine culture subsequently developed an acute post-arthroplasty infection, necessitating reintervention for debridement and surgical wound cleaning without requiring prosthesis replacement. Additionally, in a patient with a Proteus-positive urine culture, an acute surgical infection emerged, prompting reintervention and prosthesis replacement with an antibiotic spacer, followed by a revision prosthesis during the second surgery.

The identified pathogens in these patients exhibited considerable diversity. *Staphylococcus aureus* was the most prevalent, identified in twenty-eight patients [18]. Among these, three patients had a form of *Staphylococcus aureus* without resistance to any antibiotics, while the remainder either initially presented with antibiotic-resistant forms or developed resistance during treatment. Notably, one patient displayed common resistance to Penicillin, and another exhibited multiple resistances to Cefoxitin, Clindamycin, Erythromycin, Oxacillin, Penicillin, and Tetracycline [19].

Predominantly, resistance was observed solely to Penicillin; however, instances of multi-drug resistance, involving resistance to Cefoxitin, Tetracycline, Clindamycin, and Erythromycin, posed greater challenges in establishing effective antibiotic treatment.

Regarding *Staphylococcus aureus*, diverse staphylococci were also identified in these patients, including *Haemolyticus* (six cases), *Epidermidis* (six cases), *Hominis* (eight cases), *Warneri* (two cases), and *Lugdunensis* (one case).

Other microbial infections included *Enterobacter cloacae* (4 cases), *Enterococcus* (4 cases), *Enterococcus faecalis* (14 cases), *Escherichia coli* (10 cases), *Pseudomonas aeruginosa* (12 cases), *Acinetobacter baumannii* (4 cases), *Enterobacter* (8 cases), *Proteus* (6 cases), *Morganella morganii* (1 case), *Klebsiella* (10 cases), *Aerococcus viridans* (2 cases), *Proteus mirabilis* (1 case), and *Serratia marcescens* (1 case).

Among the total patient cohort, twenty-four individuals experienced infections caused by a single pathogen, while the remaining cases presented combinations of two, three, or, in isolated instances, even four pathogens during treatment. This complexity necessitated frequent adjustments to the antibiotic regimen, guided by the latest antibiogram results. For instance, among the twenty-eight patients infected with *Staphylococcus aureus*, only two exhibited exclusive infections with this microorganism, while the majority presented with concurrent infections. The distribution of microbial diversity was fairly consistent between patients with acute and chronic infections, with a notable proportion of chronic infection cases featuring the identification of a single microbe.

Within the entire cohort, a singular case emerged after a TKA, where the infection remained unresponsive to treatment efforts. Despite attempts to address the infection by removing the prosthesis and implementing an antibiotic-laden spacer, subsequent normalization of inflammatory markers prompted a knee arthroplasty. Unfortunately, the infection resurfaced post-intervention. This patient exhibited a succession of infections, initially with *Enterobacter*, followed by *Proteus* and *Staphylococcus aureus*. The latest antibiogram identified *Morganella morganii*, culminating in chronic osteomyelitis of the distal femur and proximal tibia with *Morganella morganii*.

## 4. Discussion

In the context of infection after THA, the CRP value becomes particularly significant for acute infection. A notable increase in CPR beyond normal levels is observed on day six post-surgery. The levels of CRP are four times higher than normal, with values around 111 mg/L, which should be around 26.35 mg/L. The values obtained in this group of patients are higher the in other similar studies from the literature [20].

In the case of chronic infection, the levels are slightly higher, but with no statistical significance.

After TKA, the levels of CRP in acute infection show an intriguing pattern. Despite the presumption of increases in CRP values in cases of infection, these values persist below the baseline. The CRP levels on day 1 and day 3 post-surgery are lower than expected, with a value of only 38 mg/L on day 1 and only 71 mg/L on day 3. This result is different from other studies in the literature that showed an increase in patients with acute infection after TKA [21].

In the case of chronic infection, the levels are higher, but with values similar to the increase after TKA [11].

Preoperative screening holds immense value in adequately preparing patients for THA and TKA. By collecting samples for urinary infections, pharyngeal exudate, and inguinal envelopes, surgeons can assess a patient’s overall health status and identify potential sources of infection. This proactive screening approach serves to minimize the risk of postoperative infections, contributing to the assurance that patients are in optimal condition for the impending surgical procedure. This is confirmed by other studiesin the literature [14].

The elevated prevalence of *Staphylococcus aureus*, a ubiquitous bacterium, underscores the need to address its potential impact on surgical site infections [22]. However, the fact that only a small proportion (10.71%) exhibited a non-resistant form of *Staphylococcus aureus* raises concerns, as antibiotic-resistant strains can significantly complicate treatment strategies.

A significant percentage (61.29%) of patients presenting infections with multiple germs underscores the intricate nature and complexity of managing these cases. The identification of 19 different pathogens within the infection cases highlights the vast diversity of microorganisms contributing to these infections. This diversity calls for personalized treatment plans and the capability to adapt strategies to emerging pathogens.

The strength of this study is that we followed the inflammatory samples over a long time period.

The limitations of this study are that it is a retrospective study, the number of patients enrolled in this study is small, we are not sure if all the infected patients returned to our hospital, and we did not consider other types of procedures.

## 5. Conclusions

The results of this study show that CPR postoperative values in patients with early infection after THA and TKA have higher values for THA and lower values for TKA.

Preoperative screening of asymptomatic infections is significantly related to PJI. In our group of patients, 85% of those who had a preoperative asymptomatic infection also developed a PJI. However, the pathogens identified preoperatively were identical to those responsible for PJI in 50% of the cases.

## Figures and Tables

**Table 1:** Post-THA and post-TKA inflammatory biomarkers.

Biomarker	Day	Day 1	Day 3	Day 6
CRP	THA	67.395	108.742	26.35
	TKA	80.37	191.5	65.01
ESR	THA	19.857	74.8	33
	TKA	18	91	70.85
Fibrinogen	THA	306.923	703.4	530
	TKA	378.33	603.5	621.3

**Table 2:** Post-THA and post-TKA CRP values in patients with infection.

Day	Day 1	Day 3	Day 6
THA	93.4136	115.4141	84.8823
TKA	56.5542	98.1642	79.0108

**Table 3:** CRP level in acute and chronic infections in THA.

Day	Infection Type	Mean	Std. Deviation
Day 1	acute	107.7820	37.18798
	chronic	81.4400	32.67469
Day 3	acute	128.5770	42.47206
	chronic	104.4450	32.62965
Day 6	acute	111.6820	41.10386
	chronic	62.5492	50.43827

**Table 4:** CRP levels in acute and chronic infections in TKA.

Day	Infection Type	Mean	Std. Deviation
day_1	acute	38.3120	4.18170
	chronic	69.5843	42.01109
day_3	acute	71.9860	19.57903
	chronic	116.8629	31.45554
day_6	acute	52.6900	16.51411
	chronic	97.8114	65.46878
day_21	acute	14.0660	7.12449
	chronic	32.6029	48.90507

## Data Availability

The original contributions presented in the study are included in the article, further inquiries can be directed to the corresponding author.
